# Knowledge, attitudes, and intended practices regarding colorectal polyps among patients: a cross-sectional study

**DOI:** 10.3389/fonc.2026.1711321

**Published:** 2026-05-29

**Authors:** Yongqiang Song, Yue Chen, Zhongjing Zhang, Jinming Yang, Tao Tao, Changlin Xue, Xinguo Wu, Chunyan Niu

**Affiliations:** 1 Department of Gastroenterology, Nanjing Lishui People’s Hospital, Zhongda Hospital Branch, Southest University, Nanjing, China; 2 Department of Traditional Chinese Medicine, Nanjing Lishui People’s Hospital, Zhongda Hospital Branch, Southest University, Nanjing, China; 3 Endoscopy Center of Gastroenterology, Nanjing Lishui People’s Hospital, Zhongda Hospital Branch, Southest University, Nanjing, China; 4 Department of Gastroenterology, Honglan Street Health Center, Nanjing, China; 5 Department of Gastroenterology, Baima Center Health Center, Nanjing, China

**Keywords:** colorectal polyps, cross-sectional study, knowledge, attitudes, practice, patient education, structural equation modeling

## Abstract

**Background:**

Colorectal polyps are common precancerous lesions of colorectal cancer, and effective patient self-management and secondary prevention largely depend on patients’ health-related knowledge, attitudes, and intended practices (KAP).

**Objective:**

This study aimed to assess the practice KAP regarding colorectal polyps among patients.

**Methods:**

A cross-sectional study was conducted on patients with colorectal polyps from July 2024 to December 2024 at Nanjing Lishui People’s Hospital. A self-administered questionnaire was distributed to gather demographic information and assess KAP scores.

**Results:**

A total of 472 valid responses were included in the final analysis. Among the participants, 276 (58.5%) were male, and 376 (79.7%) were newly diagnosed with intestinal polyps. The median (SD) knowledge, attitude, and intended practice scores were 5.37 (5.55) (possible range: 0-24), 36.64 (3.37) (possible range: 10-50), and 34.79 (5.32) (possible range: 9-45), respectively. Correlation analysis revealed a significant positive association between knowledge and attitude (r = 0.262, P < 0.001). Similarly, attitude and intended practices were positively correlated (r = 0.238, P < 0.001). Structural equation modeling revealed that knowledge directly influenced attitude (β = 0.589, P < 0.001) and intended practices (β = -0.334, P < 0.001). Additionally, attitude had a significant direct effect on intended practices (β = 0.508, P < 0.001). Notably, knowledge also indirectly affected intended practices through its influence on attitude (β = 0.298, P < 0.001).

**Conclusion:**

Patients with colorectal polyps exhibited limited knowledge but generally maintained positive attitudes and engaged in proactive intended practices concerning their condition. Targeted educational interventions are recommended to improve knowledge, which could lead to more informed attitudes and reinforce positive practices.

## Introduction

Colorectal polyps are common gastrointestinal lesions, representing a significant health concern worldwide (1). Contemporary estimates based on colonoscopy studies report polyp prevalence rates of 20% to 53% in individuals aged 50 years or older (1). In China specifically, studies have reported an overall polyp prevalence of 18.1%, indicating a substantial disease burden that necessitates proper medical attention and management (2). The management of colorectal polyps requires active patient participation and long-term follow-up care. Studies have shown that the 3-year cumulative recurrence rate of colorectal polyps after polypectomy ranges from 15% to 50% (3). For advanced adenomas (≥10 mm or with high-grade dysplasia), the risk of malignant transformation can be as high as 25-30% within 5-10 years if left untreated (4). Patient adherence to recommended follow-up protocols and lifestyle modifications is crucial, as studies have demonstrated that regular surveillance can reduce the risk of advanced adenomas by up to 60% (5).

The knowledge, attitudes, and practices (KAP) theory plays a pivotal role in shaping human health behaviors (6). It is often employed alongside the KAP questionnaire to comprehensively gauge the knowledge, attitude, and practices of the target population within the healthcare domain, as well as to assess the demand and level of acceptance of relevant content (7). This model, integral to health literacy, is underpinned by the fundamental premise that knowledge exerts a positive influence on attitudes, and these attitudes, in turn, shape individual practices (8).

Understanding the KAP patterns with colorectal polyps is crucial as evidence suggests that adherence to healthy lifestyle patterns and regular medical follow-up can significantly impact polyp recurrence (9). Despite the well-documented benefits of regular surveillance and lifestyle modifications, patient adherence remains suboptimal, often due to limited understanding of disease progression, fear of invasive procedures, and lack of access to healthcare resources (10). The KAP framework provides a structured approach to identifying these barriers by assessing how much patients know about colorectal polyps, their perceptions of disease severity, and their engagement in recommended health behaviors. In the field of colorectal disorders, several studies have applied the KAP framework to colorectal cancer populations, primarily focusing on patients’ or the general population’s KAP of colorectal cancer screening (11), modifiable risk factors (12, 13), or postoperative recovery (14). However, previous studies on KAP have primarily focused on colorectal cancer, behavior with very limited evidence specifically addressing patients with colorectal polyps as a distinct clinical population. This highlights a gap in the literature regarding polyp-specific KAP and underscores the need for more targeted investigations in this area. Addressing this gap is essential for developing targeted interventions aimed at enhancing patient education, improving adherence to surveillance protocols, and optimizing long-term disease management. Therefore, this study aims to assess the KAP among Chinese patients with colorectal polyps.

## Materials and methods

### Study design and participants

A cross-sectional study was conducted between July 2024 and December 2024 at Nanjing Lishui People’s Hospital, focusing on patients diagnosed with colorectal polyps. A convenience sampling method was adopted. The diagnosis of colorectal polyps followed standardized and internationally recognized criteria. Colorectal polyps were defined as epithelial neoplastic or non-neoplastic lesions confined to the mucosa or submucosa, including adenomatous polyps and serrated lesions such as hyperplastic polyps. The diagnostic criteria and classification framework were based on the 2024 European Society of Gastrointestinal Endoscopy (ESGE) Guideline (15) and the Chinese guideline for the standardized diagnosis and treatment of colorectal polyps (16). All polyps were detected by colonoscopy and subsequently confirmed through histopathological examination. Polyp assessment was performed by experienced endoscopists and gastrointestinal pathologists.

During the study period, all eligible patients with colorectal polyps who attended the hospital were consecutively invited to participate in the study. Participation was entirely voluntary, and patients independently decided whether to enroll after being informed in detail about the study objectives, procedures, and confidentiality principles. Ethical approval for the study was obtained from the Ethics Committee of Nanjing Lishui People’s Hospital (Approval No. 2024KY0607-01), and all participants provided informed consent prior to enrollment. The inclusion criteria for this study were as follows: (1) age ≥18 years and (2) ability to understand the study objectives and willingness to participate voluntarily. No specific exclusion criteria were applied in this study.

### Questionnaire

The design of the questionnaire was informed by established guidelines, specifically the *2021 Japanese Society of Gastroenterology (JSGE) Evidence-based Clinical Practice Guidelines for the Management of Colorectal Polyps* and the *Chinese expert consensus on cold snare polypectomy for colorectal polyps (2023, Hangzhou)* (17, 18). Following the development of the initial draft, expert input was solicited to enhance content accuracy and relevance, and subsequent revisions were made based on their feedback. Redundant descriptions were streamlined, professional expressions were revised, and the total number of questions was reduced. Expert consultations ensured the content validity of the questionnaire. To ensure the reliability of the instrument, a pilot survey was conducted with 44 participants, and statistical testing confirmed the questionnaire’s robustness. The overall Cronbach’s α coefficient was 0.852, indicating a robust level of internal consistency. In addition, confirmatory factor analysis (CFA) was conducted to evaluate the structural validity of the questionnaire. The model demonstrated acceptable fit indices, including the root mean square error of approximation (RMSEA = 0.069), standardized root mean square residual (SRMR = 0.085), Tucker–Lewis index (TLI = 0.894), and comparative fit index (CFI = 0.905), supporting the overall construct validity of the instrument (**Supplementary Figure 1**).

The final version of the questionnaire, administered in Chinese, consisted of four sections: demographic information, knowledge assessment, attitude evaluation, and intended practice practices assessment (Additional file 1. Questionnaire). The demographic section collected information such as age, gender, residence, educational background, average monthly household income, height, weight, history of colorectal polyps, symptoms, and family history. The knowledge section included 12 items, scored on a three-point scale: “Very familiar” (2 points), “Heard of it” (1 point), and “Not clear” (0 points), with total scores ranging from 0 to 24. The attitude section comprised 10 items, each evaluated using a five-point Likert scale, with responses ranging from “Strongly agree” (5 points) to “Strongly disagree” (1 point), yielding a total score range of 10–50. The intended practices section contained 9 items, scored on a scale from 1 (“Never”) to 5 (“Always”), resulting in a total score range of 9–45. Additionally, the final item in the intended practices section explored participants’ sources of knowledge about colorectal polyps, though it was analyzed descriptively and excluded from the scoring system. Thresholds for good knowledge, positive attitudes, and proactive behavior were defined as achieving 70.0% or higher of the total score (19).

### Questionnaire distribution and quality control

The study employed a mixed-mode questionnaire-based approach, utilizing both electronic surveys administered through the Questionnaire Star platform and traditional paper-based questionnaires for data collection. All data were collected quantitatively using structured questionnaires, and no qualitative interviews or qualitative analyses were conducted. Convenient sampling was implemented to select participants, and the questionnaires were distributed in person by members of the research team within outpatient clinics and hospital wards. During the data collection process, the researchers provided on-site guidance to participants, addressing any questions or concerns to ensure clarity and accuracy. Completed questionnaires were retrieved directly by the research team to maintain control over the data collection process.

To further ensure data integrity, a research assistant, who received specialized training from the hospital’s Academic Committee, Ethics Committee, and Department of Scientific Research and Education, was designated to oversee the collection, entry, and organization of the data. This process included rigorous data quality control measures, which excluded responses based on the following criteria: a completion time of less than 90 seconds, abnormal or incomplete information, or logical inconsistencies identified through embedded quality control questions. The questionnaire also included a general knowledge “trap” question. Incorrect answers to this trap question were considered as invalid responses, indicating that the participant did not carefully review the question before answering.

### Sample size calculation

The sample size was calculated using the formula for cross-sectional studies (20): n = Z²P(1-P)/E², where Z represents the Z-value for the desired confidence level (1.96 for 95% confidence), P represents the estimated proportion (0.5), and E represents the margin of error (0.05). In the sample size estimation, the assumed proportion (P) was set at 0.50 due to the absence of previously published KAP studies focusing on colorectal polyps. This value was chosen as it yields the maximum variability (P(1−P)) and therefore results in the largest required sample size, providing a conservative estimate that ensures adequate statistical power. The minimum required sample size was calculated to be 385 participants. To account for potential incomplete responses and dropouts, we increased the target enrollment by approximately 20%, and the final sample size was set to 480.

### Statistical analysis

The normality of continuous data was assessed using the Kolmogorov-Smirnov test. Continuous variables all followed a skewed distribution analyze. For variables with a skewed distribution, data were presented as medians (ranges) and analyzed using the Wilcoxon-Mann-Whitney U-test or the Kruskal-Wallis test. Categorical variables were described as frequencies (n, %). Correlations between scores across different dimensions were assessed using Spearman’s correlation coefficients. Structural equation modeling (SEM), guided by the KAP theoretical framework, was employed to explore whether attitudes mediated the relationship between knowledge and intended practices. Both direct and indirect effects were computed and compared. The knowledge dimension of the questionnaire consisted of 12 items, which were classified into four subdomains: Basic Knowledge (K1, K3, K4), Risk Factor Knowledge (K2, K5), Symptoms & Diagnosis (K6-K7), and Treatment & Prognosis Knowledge (K8-K12). The attitude dimension comprised 10 items and was subdivided into four subdomains: Perceived Benefits (A1-A2), Medical Trust & Adherence (A3-A4), Risk Perception (A5-A7, A10), and Treatment Decision (A8-A9). These subdomains were subsequently incorporated as latent variables in the SEM analysis to examine their relationships with practice intended practices. The model’s goodness-of-fit was evaluated using the following criteria: RMSEA and SRMR values below 0.08, and TLI and CFI values greater than 0.8. Statistical significance was defined as a two-tailed P value < 0.05. All analyses were performed using Stata version 18.0 (Stata Corp LLC, College Station, TX, USA) and R version 4.3.2 (R Foundation for Statistical Computing, Vienna, Austria).

## Results

A total of 515 questionnaires were collected. Invalid responses were excluded: 3 questionnaires with completion times under 90 seconds, 6 cases with abnormal values for age, weight, or height, and 34 questionnaires containing logical errors in trap question. After excluding these invalid responses, the final dataset consisted of 472 valid responses.

### Basic information on the population

Among them, 276 (58.5%) were male, 268 (56.8%) were aged 45-64 years, 224 (47.5%) had middle school education or below, 307 (65.0%) had a stable income, 376 (79.7%) were first found to have intestinal polyps, and only 181 (38.3%) had received education about colorectal from a hospital. The median (SD) knowledge, attitude, and intended practice scores were 5.37 (5.55) (possible range: 0-24), 36.64 (3.37) (possible range: 10-50), and 34.79 (5.32) (possible range: 9-45), respectively. Analyses of demographic characteristics found that participants’ knowledge, attitude, and practice scores varied across age, residence, education, type of income, household’s average monthly income, first time of colorectal polyps, and education about colorectal from a hospital (all P < 0.05). Meanwhile, knowledge scores also varied significantly by hyperlipidemia status (P = 0.033), constipation status (P = 0.02), fatty liver status (P < 0.001), and family members with colorectal polyps (P < 0.001). Attitude scores varied significantly by diabetes status (P = 0.039). Practice scores varied significantly by gender (P = 0.001), hyperlipidemia status (P < 0.001), diabetes status (P < 0.001), constipation status (P < 0.001), fatty liver status (P = 0.001), and family members with colorectal polyps (P < 0.001) (**Table 1**). In addition, proportion-based analyses of knowledge, attitude, and intended practices scores across demographic groups are presented in **Supplementary Table 1** to provide a more intuitive comparison of group differences.

**Table 1:** Baseline characteristics and the KAP scores of the patients.

N=472	N(%)	Knowledge	P	Attitude	P	Practice	P
		median [IQR]		median [IQR]		median [IQR]	
Total score	472(100.0)	4.00 [0.00, 10.00]		36.00 [34.00, 38.00]		35.00 [32.00, 38.00]	
Gender			0.985		0.378		0.001
Male	276(58.5)	4.00 [0.00, 9.25]		36.00 [34.00, 38.00]		34.00 [30.00, 38.00]	
Female	196(41.5)	5.00 [0.00, 10.00]		37.00 [34.75, 39.00]		36.00 [33.00, 38.00]	
Age			<0.001		0.009		0.033
19-44 years	75(15.9)	10.00 [5.00, 12.50]		37.00 [35.50, 39.00]		36.00 [34.00, 38.00]	
45-64 years	268(56.8)	4.00 [0.00, 9.00]		36.00 [35.00, 38.25]		35.00 [32.00, 38.00]	
65 years or more	129(27.3)	0.00 [0.00, 7.00]		36.00 [34.00, 38.00]		34.00 [31.00, 37.00]	
Residence			<0.001		0.003		<0.001
Rural	206(43.6)	1.00 [0.00, 7.00]		36.00 [34.00, 38.00]		34.00 [30.25, 37.00]	
Urban	266(56.4)	6.00 [0.00, 11.00]		37.00 [35.00, 39.00]		36.00 [33.00, 38.00]	
Education			<0.001		0.006		0.004
Middle school or below	224(47.5)	1.00 [0.00, 6.00]		36.00 [34.00, 38.00]		34.00 [31.00, 37.00]	
High school/technical school	107(22.7)	7.00 [0.00, 10.00]		37.00 [35.00, 39.00]		35.00 [32.50, 38.00]	
Associate degree	54(11.4)	9.00 [2.25, 11.75]		36.00 [35.00, 38.00]		36.00 [33.00, 38.00]	
Bachelor’s degree or above	87(18.4)	6.00 [0.00, 12.00]		37.00 [35.00, 40.00]		36.00 [34.00, 38.00]	
Type of income			<0.001		0.001		<0.001
Stable income	307(65.0)	6.00 [0.00, 11.00]		36.00 [35.00, 39.00]		36.00 [33.00, 39.00]	
Unstable income	165(35.0)	0.00 [0.00, 7.00]		36.00 [34.00, 38.00]		33.00 [30.00, 36.00]	
Household’s average monthly income (CNY)			<0.001		<0.001		<0.001
<2000	93(19.7)	0.00 [0.00, 6.00]		35.00 [33.00, 38.00]		33.00 [30.00, 35.00]	
2000-5000	161(34.1)	4.00 [0.00, 9.00]		36.00 [34.00, 38.00]		36.00 [32.00, 40.00]	
5000-10000	134(28.4)	8.00 [2.00, 11.00]		37.00 [35.00, 39.00]		36.00 [33.00, 38.00]	
>20000	84(17.8)	4.00 [0.00, 8.25]		37.00 [35.00, 39.00]		36.00 [33.75, 38.00]	
First time of colorectal polyps			<0.001		0.011		<0.001
Yes	376(79.7)	2.00 [0.00, 8.00]		36.00 [34.00, 38.00]		35.50 [32.00, 38.00]	
No	96(20.3)	9.00 [6.00, 11.00]		37.00 [35.00, 39.00]		33.00 [28.00, 37.00]	
Hyperlipidemia			0.033		0.888		<0.001
No	409(86.7)	4.00 [0.00, 9.00]		36.00 [35.00, 38.00]		35.00 [32.00, 38.00]	
Yes	63(13.3)	7.00 [2.00, 10.00]		37.00 [34.00, 39.00]		32.00 [27.00, 36.00]	
Diabetes			0.103		0.039		<0.001
No	344(72.9)	4.00 [0.00, 10.00]		36.00 [35.00, 39.00]		36.00 [33.00, 38.00]	
Yes	128(27.1)	4.00 [0.00, 8.00]		36.00 [34.00, 38.00]		34.00 [30.00, 36.25]	
Constipation			0.020		0.415		<0.001
No	428(90.7)	4.00 [0.00, 10.00]		36.00 [35.00, 38.00]		35.00 [32.00, 38.00]	
Yes	44(9.3)	7.50 [3.50, 9.00]		36.00 [34.00, 38.25]		32.50 [27.50, 35.00]	
Fatty liver			<0.001		0.1		0.001
No	413(87.5)	4.00 [0.00, 9.00]		36.00 [35.00, 38.00]		35.00 [32.00, 38.00]	
Yes	59(12.5)	8.00 [3.50, 12.00]		37.00 [34.00, 40.00]		33.00 [28.00, 36.50]	
Family members with colorectal polyps			<0.001		0.095		<0.001
No	301(63.8)	0.00 [0.00, 6.00]		36.00 [34.00, 38.00]		36.00 [33.00, 38.00]	
Yes	171(36.2)	9.00 [6.00, 11.00]		36.00 [35.00, 39.00]		34.00 [30.00, 37.00]	
Education about colorectal from a hospital			<0.001		<0.001		0.001
No	291(61.7)	0.00 [0.00, 4.00]		36.00 [34.00, 38.00]		36.00 [33.00, 38.00]	
Yes	181(38.3)	10.00 [8.00, 12.00]		37.00 [35.00, 40.00]		34.00 [30.00, 38.00]	

All continuous variables in this table were assessed for normality and found to be skewed distributed; therefore, data are presented as median (interquartile range), and group comparisons were conducted using the Wilcoxon-Mann-Whitney U-test or Kruskal-Wallis test as appropriate. Data are presented as median (interquartile range), and group comparisons were conducted using the Wilcoxon-Mann-Whitney U-test or Kruskal-Wallis test as appropriate.

### Distribution of responses to knowledge, attitude, and intended practices

The distribution of knowledge dimensions showed that the three questions with the highest number of participants choosing the “Not clear “ option were “Colorectal polyps can occur in any part of the intestine, with the left colon being the most common site.” (K4) with 88.77%, “The incidence of colorectal polyps is higher in males than in females.” (K2) with 83.05%, and “Colorectal polyps refer to protruding lesions on the surface of the intestinal mucosa into the intestinal cavity, characterized as abnormally growing tissues.” (K1) with 64.62% (**Table 2**; **Supplementary Figure 2**).

**Table 2:** Knowledge dimension distribution. .

Items, n (%)	Very familiar	Heard of it	Not clear
1.Colorectal polyps refer to protruding lesions on the surface of the intestinal mucosa into the intestinal cavity, characterized as abnormally growing tissues.	29(6.14%)	138(29.24%)	305(64.62%)
2.The incidence of colorectal polyps is higher in males than in females.	6(1.27%)	74(15.68%)	392(83.05%)
3.Colorectal polyps include both neoplastic and non-neoplastic lesions.	29(6.14%)	173(36.65%)	270(57.2%)
4.Colorectal polyps can occur in any part of the intestine, with the left colon being the most common site.	6(1.27%)	47(9.96%)	419(88.77%)
5.The occurrence of colorectal polyps is associated with various factors, including genetic predisposition, unhealthy lifestyle habits, dietary habits, smoking, alcohol consumption, obesity, and diabetes.	18(3.81%)	153(32.42%)	301(63.77%)
6.Most patients with colorectal polyps exhibit no obvious symptoms, while a minority may experience symptoms such as rectal bleeding, hematochezia, or changes in bowel habits.	17(3.6%)	163(34.53%)	292(61.86%)
7.A pathological biopsy is required to determine the nature of the polyps and assess whether they are neoplastic.	24(5.08%)	178(37.71%)	270(57.2%)
8.In the absence of contraindications, endoscopic surgical treatment is the preferred approach for managing colorectal polyps.	59(12.5%)	177(37.5%)	236(50%)
9.Once detected, colorectal polyps should be treated promptly to prevent their progression to cancerous polyps.	62(13.14%)	196(41.53%)	214(45.34%)
10.Patients with colorectal polyps should maintain a healthy lifestyle, avoid smoking and alcohol, consume a light diet, and reduce the intake of high-fat, high-oil foods.	25(5.3%)	208(44.07%)	239(50.64%)
11.Over 90% of colorectal cancers originate from polyps, and the larger the polyp, the greater the risk of malignant transformation.	21(4.45%)	176(37.29%)	275(58.26%)
12.The removal of colorectal polyps via endoscopy is considered curative, but there is a risk of recurrence after treatment.	30(6.36%)	201(42.58%)	241(51.06%)

Responses to the attitude dimension showed that 9.32% strongly agreed and 27.33% agreed that colorectal polyps are not a serious health problem (A5), 2.97% were very worried and 41.53% worried that the polyps may recur (A10), and 3.39% very worried and 29.66% worried that the polyps may develop into colorectal cancer in the future (A7) (**Table 3**; **Supplementary Figure 3**).

**Table 3:** Attitude dimension distribution.

Items, n (%)	Strongly agree	Agree	Neutral	Disagree	Strongly disagree
1. I believe that regular colonoscopy examinations are highly necessary.	136 (28.81%)	266 (56.36%)	68 (14.41%)	0 (0%)	2 (0.42%)
2. I believe that modifying lifestyle and dietary habits can reduce the risk of colorectal polyps.	109 (23.09%)	235 (49.79%)	127 (26.91%)	1 (0.21%)	0 (0%)
3. I trust the treatment plan prescribed by my doctor.	178 (37.71%)	279 (59.11%)	15 (3.18%)	0 (0%)	0 (0%)
4. I am willing to follow my doctor’s advice and undergo regular follow-up examinations after polyp removal to check for recurrence.	154 (32.63%)	286 (60.59%)	30 (6.36%)	1 (0.21%)	1 (0.21%)
5. I believe that colorectal polyps are not a serious health problem.	44 (9.32%)	129 (27.33%)	226 (47.88%)	61 (12.92%)	12 (2.54%)
6. I am concerned that my polyps may be neoplastic in nature.	17 (3.6%)	127 (26.91%)	237 (50.21%)	59 (12.5%)	32 (6.78%)
7. I am worried that the polyps may develop into colorectal cancer in the future.	16 (3.39%)	140 (29.66%)	220 (46.61%)	62 (13.14%)	34 (7.2%)
8. I am willing to undergo surgical treatment for colorectal polyps as soon as possible.	145 (30.72%)	278 (58.9%)	44 (9.32%)	1 (0.21%)	4 (0.85%)
9. I am concerned about the risks associated with surgery.	15 (3.18%)	105 (22.25%)	249 (52.75%)	65 (13.77%)	38 (8.05%)
10. I am worried that the polyps may recur.	14 (2.97%)	196 (41.53%)	219 (46.4%)	17 (3.6%)	26 (5.51%)

Responses to the intended practices dimension showed that 2.12% rarely and 10.38% never try quit smoking (P2.4), 1.48% rarely and 10.81% never limit alcohol consumption (P2.5), 27.97% rarely and 0.85% never actively encourage those around me to prevent and screen for colorectal polyps (P3). When it comes to the channels to learn about knowledge related to colorectal polyps (P4), the most reported were hospital campaigns, attending physician, or other doctors’ education (80.3%), followed by conversations with relatives, friends, or fellow patients (58.47%) (**Table 4**; **Supplementary Figure 4**).

**Table 4:** Practice dimension distribution. .

Practice	Always	Often	Sometimes	Rarely	Never
1. After surgical removal of polyps, I will undergo regular follow-up colonoscopies.	98 (20.76%)	256 (54.24%)	114 (24.15%)	4 (0.85%)	0 (0%)
2. In daily life, I will pay attention to the following:					
2.1 Maintain a balanced diet to ensure adequate nutrient intake.	68 (14.41%)	313 (66.31%)	90 (19.07%)	1 (0.21%)	0 (0%)
2.2 Consume more vegetables, fruits, and other foods rich in fiber.	84 (17.8%)	313 (66.31%)	67 (14.19%)	8 (1.69%)	0 (0%)
2.3 Follow a light diet and avoid high-fat, high-sugar, and overly stimulating foods.	82 (17.37%)	258 (54.66%)	101 (21.4%)	30 (6.36%)	1 (0.21%)
2.4 Quit smoking.	328 (69.49%)	24 (5.08%)	61 (12.92%)	10 (2.12%)	49 (10.38%)
2.5 Limit alcohol consumption.	330 (69.92%)	26 (5.51%)	58 (12.29%)	7 (1.48%)	51 (10.81%)
2.6 Develop a habit of regular bowel movements.	99 (20.97%)	308 (65.25%)	62 (13.14%)	2 (0.42%)	1 (0.21%)
2.7 Maintain regular physical exercise.	69 (14.62%)	144 (30.51%)	142 (30.08%)	109 (23.09%)	8 (1.69%)
3.I will actively encourage those around me to prevent and screen for colorectal polyps.	65 (13.77%)	118 (25%)	153 (32.42%)	132 (27.97%)	4 (0.85%)

### Correlations between KAP

Correlation analysis indicated significant positive correlations between knowledge and attitude (r = 0.262, P < 0.001). Meanwhile, there was also a positive correlation between attitude and intended practices (r = 0.238, P < 0.001) (**Table 5**).

**Table 5:** Correlation analysis.

Spearman	Knowledge	Attitude	Practice
Knowledge	1.000		
Attitude	0.262 (P<0.001)	1.000	
Practice	-0.001 (P = 0.990)	0.238 (P<0.001)	1.000

### SEM analysis

**Figure 1:**
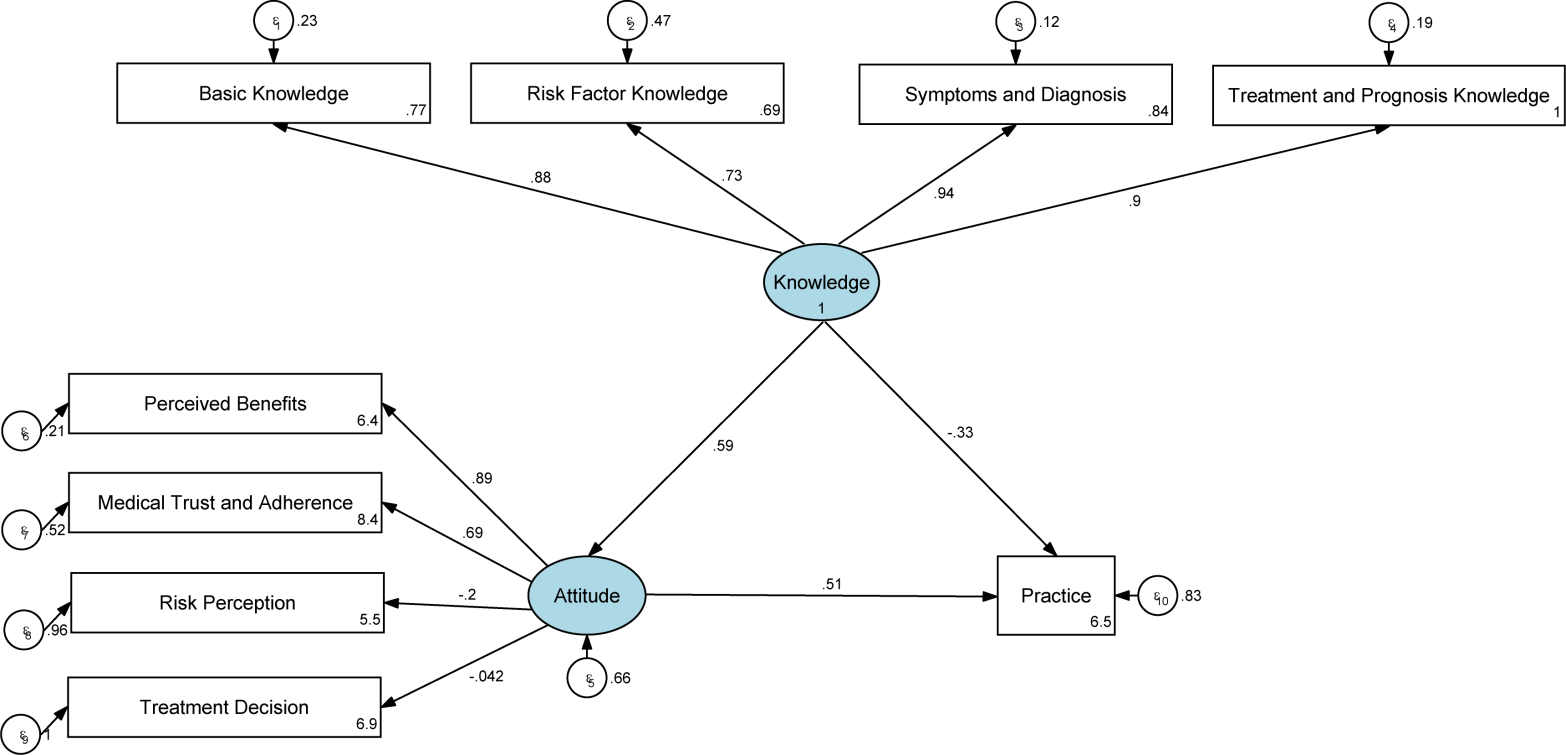
SEM model.

The SEM demonstrated a favor acceptable indices (RMSEA value: 0.069, SRMR value: 0.085, TLI value: 0.894, and CFI value: 0.905) (**Supplementary Table 2**). The results revealed that knowledge had a significant direct effect on attitude (β = 0.586, P < 0.001) but a negative direct effect on intended practices (β = -0.334, P < 0.001). Attitude, in turn, exerted a strong positive direct effect on intended practices (β = 0.508, P < 0.001). Notably, knowledge also indirectly influenced practice through attitude (β = 0.298, P < 0.001) (**Supplementary Table 3**; **Figure 1**).

## Discussion

Patients with colorectal polyps demonstrated insufficient knowledge but generally positive attitudes and proactive practice intended practices toward managing their condition. Targeted educational interventions focusing on enhancing knowledge about colorectal polyps may further improve patient attitudes and intended practices, ultimately supporting better disease prevention and management strategies.

The findings of this study reveal critical gaps in knowledge among patients with colorectal polyps, despite their generally positive attitudes and proactive practice intended practices. Similar studies have highlighted that knowledge about the causes, risks, and prevention of colorectal polyps is often lacking, particularly in populations with lower levels of education or access to health information (21, 22). However, the positive attitudes and reported adherence to recommended practices align with findings from research emphasizing the role of healthcare providers and structured clinical environments in shaping patient behavior (23, 24). These patterns suggest that while patients may rely heavily on their doctors for guidance, broader efforts to improve public awareness and self-efficacy remain insufficiently addressed.

The relationship between knowledge, attitude, and intended practices, as revealed by the correlation and structural equation modeling analyses, provides further insight into these dynamics. Although the model fit indices did not fully meet the most stringent recommended thresholds, particularly with SRMR slightly exceeding the conventional cutoff, the overall pattern of results remains interpretable and meaningful within the context of this study. It is important to note that SEM fit indices should be evaluated comprehensively rather than based on a single criterion, and acceptable values of TLI and CFI in this study support the overall adequacy of the model. In addition, the complexity of the measurement structure, including multiple observed variables across KAP domains, may have contributed to the marginal deviation in certain fit indices. Despite these limitations, the identified pathways are consistent with the theoretical KAP framework and align with existing literature, suggesting that the model retains practical and clinical relevance in understanding patient behavioral intentions. The significant association between knowledge and attitude underscores the foundational role of education in fostering positive perspectives toward health management, as similarly demonstrated in studies on other chronic conditions (25). Moreover, the indirect influence of knowledge on intended practices, mediated through attitude, reinforces the idea that knowledge alone is not sufficient to drive behavior change. This finding aligns with the broader literature, which has consistently shown that attitudes act as a crucial intermediary, translating understanding into actionable practices (26, 27). Interestingly, the direct negative effect of knowledge on intended practices observed in this study, together with the near-zero correlation between knowledge and intended practices, suggests a more complex relationship that may not fully align with the traditional linear assumptions of the KAP model. One possible explanation is that higher levels of knowledge may increase patients’ awareness of disease severity, recurrence risk, or potential complications, which in turn may lead to anxiety, uncertainty, or avoidance tendencies rather than proactive behavioral intentions, a phenomenon documented in other health behavior research practice behavior (28). In addition, the intended practices measured in this study reflect behavioral intentions rather than actual long-term behaviors, which may be influenced by external factors such as physician guidance, healthcare accessibility, or social support, thereby weakening the direct association with knowledge. Another possible explanation is that the measurement dimensions of knowledge and intended practices may capture partially different constructs, resulting in an inconsistent direct relationship. Despite this, the positive indirect pathway through attitude indicates that knowledge still plays a meaningful role in shaping intended practices via attitudinal changes, which remains consistent with the broader KAP framework. This negative association might be explained by psychological factors - increased knowledge about disease complications could potentially trigger anxiety or avoidance behaviors, a phenomenon that has been observed in previous studies of preventive health behaviors (29).

The disparities in KAP scores across various demographic and socioeconomic groups underscore persistent structural inequities in healthcare delivery and education. Individuals residing in urban areas, those with higher income levels, and those possessing greater educational attainment consistently demonstrated significantly superior KAP scores, a trend consistent with findings from studies conducted in both high-income and low- to middle-income countries (30). These inequalities emphasize the profound influence of differential access to healthcare resources, such as educational initiatives, routine health screenings, and physician engagement. Moreover, participants who had previously received information about colorectal polyps from healthcare professionals exhibited substantially higher scores across all KAP dimensions, highlighting the pivotal role of targeted educational interventions in bridging knowledge gaps and enhancing health outcomes (31, 32).

The detailed analysis of individual KAP items provides further clarity on specific areas that require intervention. Most participants demonstrated limited understanding of fundamental concepts, such as the risk factors for colorectal polyps and the importance of prompt treatment. These deficiencies are consistent with global studies that have identified a lack of emphasis on disease-specific education in routine clinical settings (33, 34). Moreover, while attitudes toward preventive measures, such as colonoscopy screenings, were generally positive, concerns about potential disease severity and surgical risks were prevalent, suggesting the need for improved communication strategies to address patient fears and misconceptions (35, 36). In terms of practice, adherence to follow-up recommendations and dietary adjustments was relatively high, but gaps in other areas, such as physical activity and proactive health advocacy, indicate opportunities for further improvement. These findings align with global research showing that while patients may adopt some recommended practices, sustained behavioral changes often require ongoing support and reinforcement (37).

Addressing these gaps will require a more structured and targeted approach. Efforts to enhance patient knowledge should include the integration of evidence-based educational materials into routine healthcare interactions, with a focus on key content areas identified in this study, such as the common anatomical distribution of colorectal polyps, major risk factors (including diet, smoking, alcohol use, obesity, and metabolic conditions), the potential for malignant transformation, and the importance of timely treatment and regular surveillance. Educational materials should be simplified, visualized, and tailored to individuals with lower educational levels to improve comprehension and retention. For instance, healthcare providers could implement structured counseling sessions during clinic visits, supported by multimedia resources and follow-up materials, to reinforce understanding and promote adherence to recommended practices. Community-based outreach programs could also play a pivotal role in bridging knowledge gaps, particularly in rural and underserved areas, by leveraging local networks and culturally appropriate messaging to enhance engagement and accessibility practice program (38, 39). In addition, integrating education into primary healthcare services and utilizing mobile health platforms may further improve reach and continuity of care. Beyond patient-level interventions, systemic changes are necessary to ensure that healthcare providers are adequately trained to deliver consistent and effective education. This may include professional development programs focused on communication skills and patient education strategies, as well as incentives for clinicians to prioritize preventive care.

Healthcare organizations could also explore innovative solutions, such as telemedicine platforms and mobile health applications, to disseminate information and support patients in managing their conditions. These tools have been shown to improve knowledge retention and facilitate sustained behavioral changes in other healthcare contexts, and their adaptation to colorectal health education could yield similar benefits (40). Furthermore, policies aimed at reducing socioeconomic barriers to care, such as subsidized screening programs and public health campaigns, could help address the disparities observed in this study.

Based on our findings, targeted prevention and health promotion strategies are essential for patients with colorectal polyps. Educational interventions should prioritize enhancing knowledge of polyp malignancy risk, modifiable lifestyle factors, and the importance of regular surveillance, as improved knowledge is foundational for sustaining positive attitudes and preventive behaviors (11, 12, 41). Clinician-led counseling during routine clinical encounters, combined with written or digital educational materials, may effectively reinforce adherence to follow-up and risk-reduction practices (42). In addition, lifestyle-focused health promotion - emphasizing healthy diet, physical activity, smoking cessation, and alcohol moderation - should be integrated into patient management, as these behaviors are closely associated with reduced colorectal neoplasia risk. System-level and community-based interventions, particularly for underserved populations, alongside strengthened physician–patient communication, may further reduce disparities and enhance secondary prevention outcomes.

This study has several limitations that should be acknowledged. The use of a convenience sampling method and recruitment from a single center may introduce selection bias, as the study population may not be fully representative of patients with colorectal polyps in other regions or healthcare settings with different levels of medical resources. First, as a cross-sectional study, it cannot establish causal relationships between knowledge, attitudes, and intended practices, thereby limiting the ability to infer temporal sequences. Second, the use of a self-administered questionnaire may have introduced response bias, as participants might overestimate their attitudes or intended practices. Third, the study was conducted in a single hospital, which may restrict the generalizability of the findings to broader populations with varying demographic and cultural contexts. In addition, although structural equation modeling was applied to explore the relationships among KAP dimensions, the cross-sectional design still limits the ability to establish causal inferences. Furthermore, the use of self-reported questionnaires may introduce social desirability bias, as participants may overreport favorable attitudes or intended practices. Finally, important factors such as patients’ health literacy levels and other potential confounding variables were not assessed in this study, which may influence the interpretation of the findings.

In conclusion, patients with colorectal polyps demonstrated inadequate knowledge but maintained positive attitudes and proactive practices regarding their condition, highlighting the influence of attitude as a mediating factor between knowledge and practice. Targeted educational interventions aimed at improving patients’ knowledge about colorectal polyps are essential, as this could enhance both attitudes and practical behaviors, ultimately contributing to better disease management and prevention strategies.

## Data Availability

The original contributions presented in the study are included in the article/**Supplementary Material**. Further inquiries can be directed to the corresponding author/s.

## Supplementary material

Supplementary Figure 1 CFA model.

Supplementary Figure 2 Distribution of responses in the knowledge dimension.

Supplementary Figure 3 Distribution of responses in the attitude dimension.

Supplementary Figure 4 Distribution of responses in the intended practices dimension.
