# Supplementary figures and images for: Knowledge, attitudes, and intended practices regarding colorectal polyps among patients: a cross-sectional study
Source: Front Oncol. 2026 May 29;16:1711321. doi: 10.3389/fonc.2026.1711321 (PMC13247643; doi:10.3389/fonc.2026.1711321)

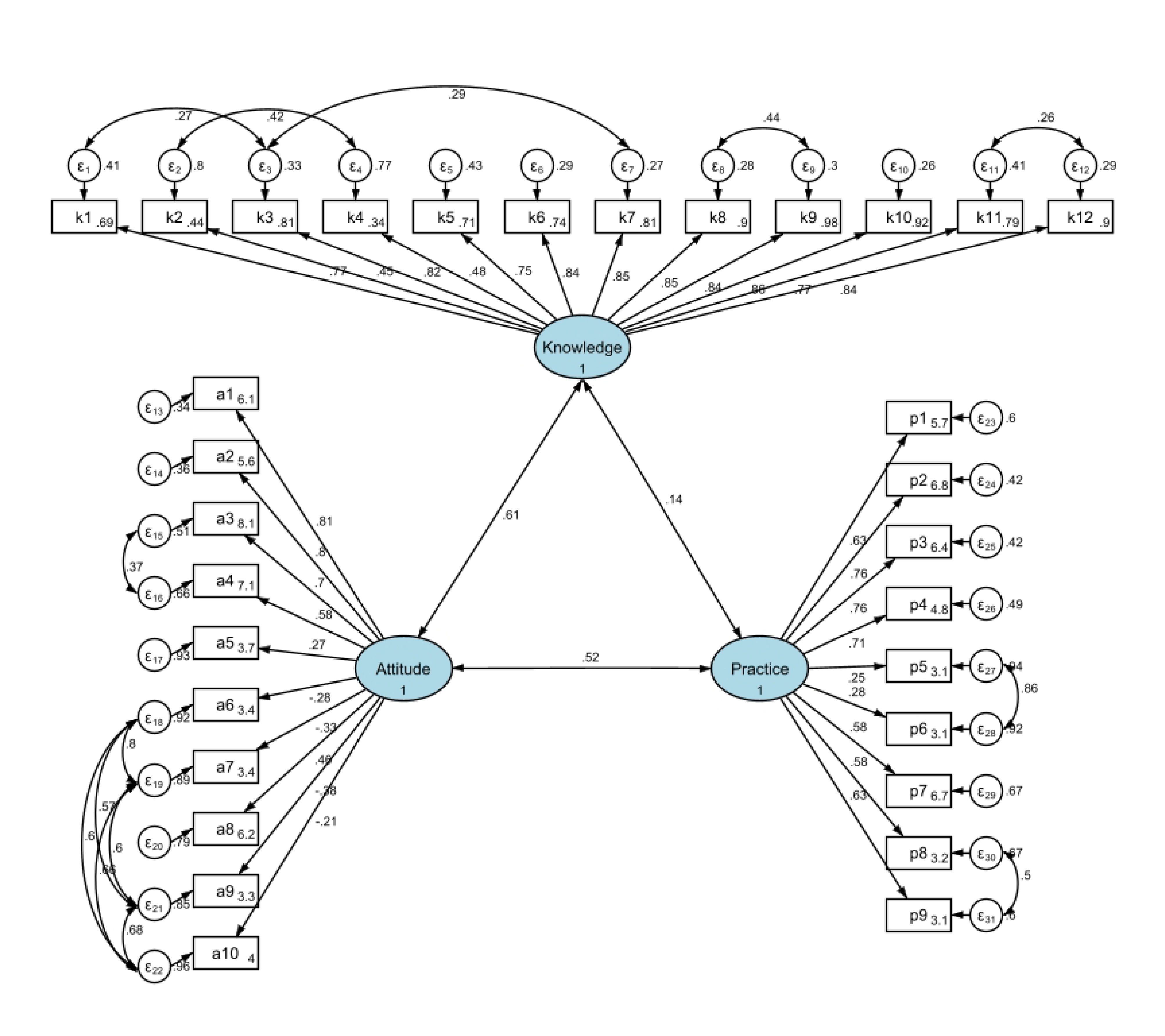

Supplement: Supplementary Figure 1 — CFA model. [file Image1.tif]

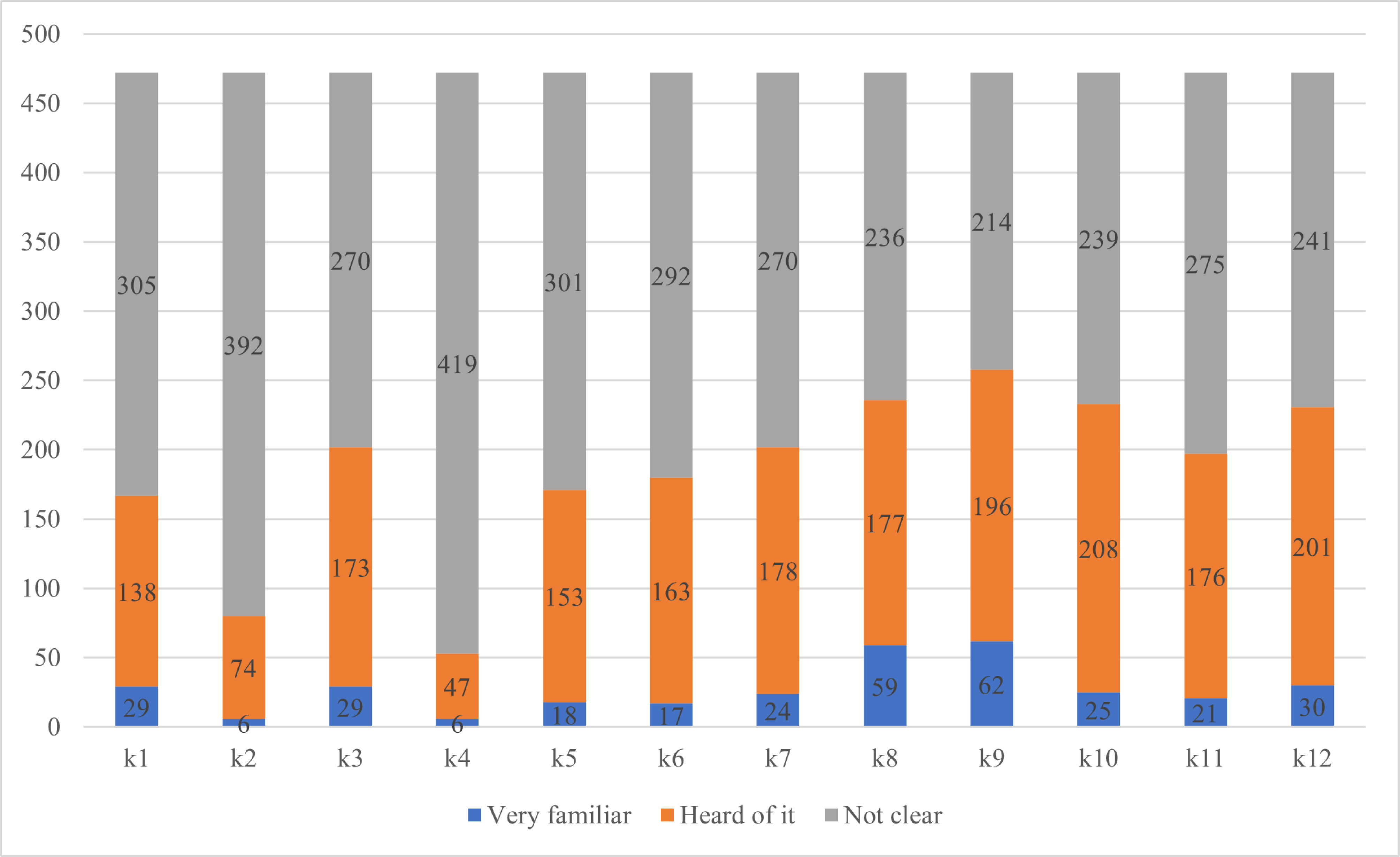

Supplement: Supplementary Figure 2 — Distribution of responses in the knowledge dimension. [file Image2.tif]

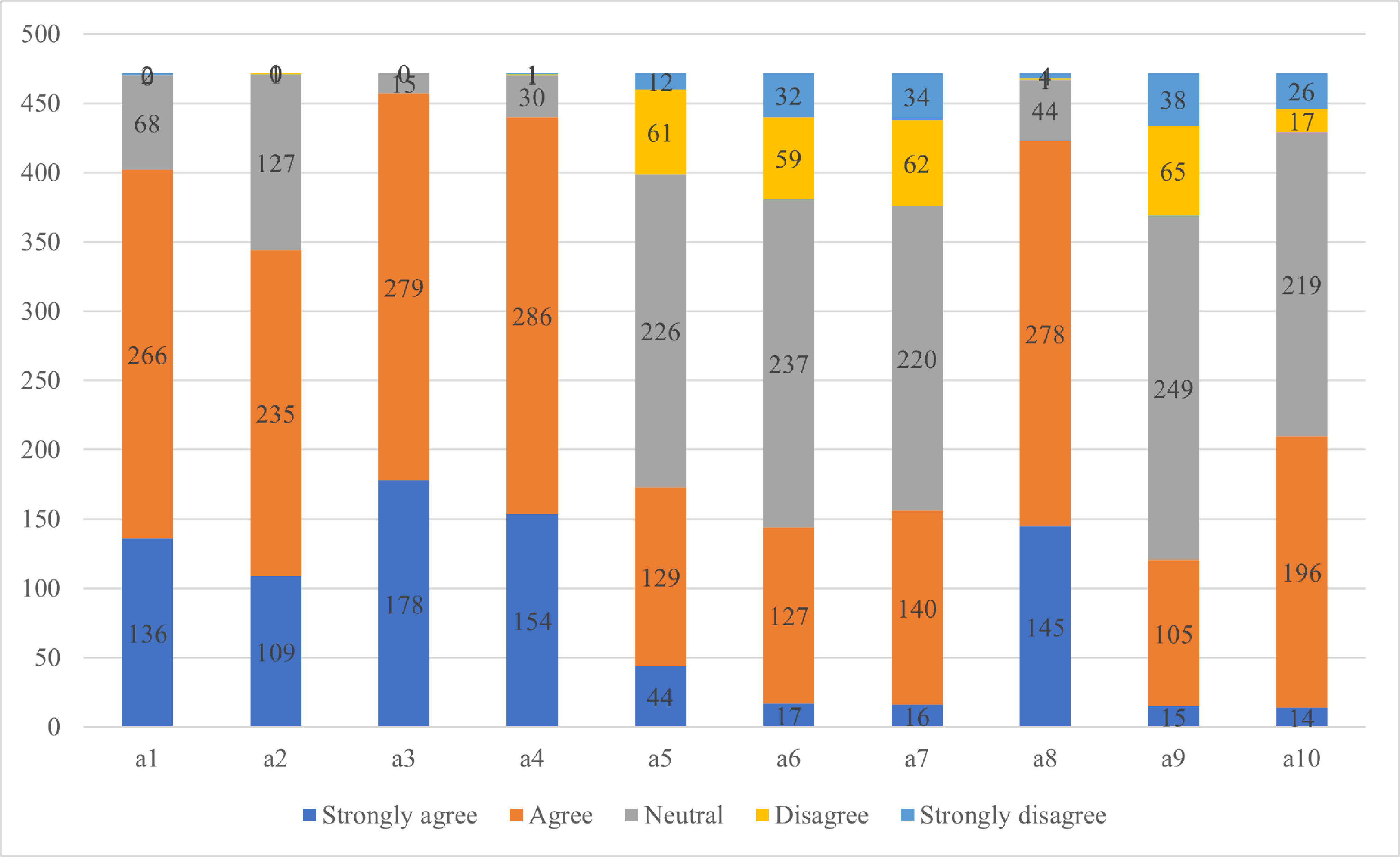

Supplement: Supplementary Figure 3 — Distribution of responses in the attitude dimension. [file Image3.tif]

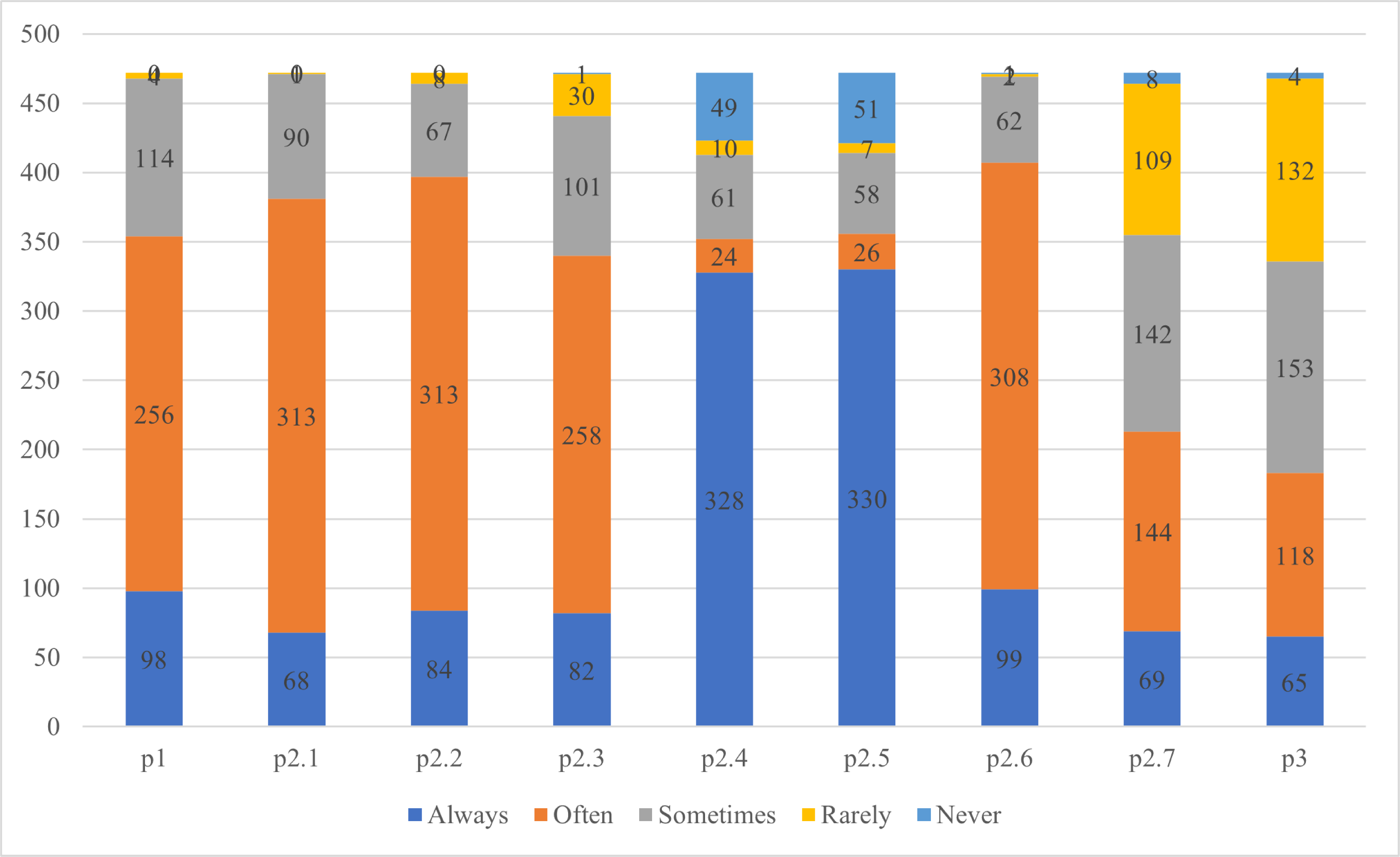

Supplement: Supplementary Figure 4 — Distribution of responses in the intended practices dimension. [file Image4.tif]
