# Supplementary material for: Knowledge, attitudes, and intended practices regarding colorectal polyps among patients: a cross-sectional study
Source: Front Oncol. 2026 May 29;16:1711321. doi: 10.3389/fonc.2026.1711321 (PMC13247643; doi:10.3389/fonc.2026.1711321)
Supplement: Supplementary file 5 [file Table1.docx]

**Table S1. Proportion-based distribution of knowledge, attitude, and intended practices scores across different demographic characteristics**

| **N=472** | N（%） | Knowledge | P | Attitude | P | Practice | P |
| --- | --- | --- | --- | --- | --- | --- | --- |
|  |  | Median [IQR] |  | Median [IQR] |  | Median [IQR] |  |
| **Total score** | 472(100.0) | 0.04 (0.20) |  | 0.75 (0.44) |  | 0.78 (0.41) |  |
| **Gender** |  |  | 0.433 |  | 0.859 |  | <0.001 |
| Male | 276(58.5) | 0.04 (0.19) |  | 0.74 (0.44) |  | 0.72 (0.45) |  |
| Female | 196(41.5) | 0.05 (0.22) |  | 0.75 (0.43) |  | 0.87 (0.34) |  |
| **Age** |  |  | <0.001 |  | 0.002 |  | 0.190 |
| 19-44 years | 75(15.9) | 0.13 (0.34) |  | 0.85 (0.36) |  | 0.85 (0.36) |  |
| 45-64 years | 268(56.8) | 0.03 (0.18) |  | 0.76 (0.42) |  | 0.78 (0.41) |  |
| 65 years or more | 129(27.3) | 0.01 (0.09) |  | 0.64 (0.48) |  | 0.74 (0.44) |  |
| **Residence** |  |  | <0.001 |  | 0.001 |  | <0.001 |
| Rural | 206(43.6) | 0.00 (0.07) |  | 0.67 (0.47) |  | 0.69 (0.46) |  |
| Urban | 266(56.4) | 0.07 (0.26) |  | 0.80 (0.40) |  | 0.85 (0.35) |  |
| **Education** |  |  | <0.001 |  | 0.001 |  | 0.041 |
| Middle school or below | 224(47.5) | 0.00 (0.00) |  | 0.67 (0.47) |  | 0.73 (0.44) |  |
| High school/technical school | 107(22.7) | 0.05 (0.21) |  | 0.78 (0.42) |  | 0.81 (0.39) |  |
| Associate degree | 54(11.4) | 0.07 (0.26) |  | 0.83 (0.38) |  | 0.80 (0.41) |  |
| Bachelor’s degree or above | 87(18.4) | 0.13 (0.33) |  | 0.86 (0.35) |  | 0.87 (0.33) |  |
| **Type of income** |  |  | 0.004 |  | <0.001 |  | <0.001 |
| Stable income | 307(65.0) | 0.06 (0.24) |  | 0.81 (0.39) |  | 0.85 (0.36) |  |
| Unstable income | 165(35.0) | 0.01 (0.08) |  | 0.63 (0.48) |  | 0.66 (0.47) |  |
| **Household’s average monthly income (CNY)** |  |  | 0.001 |  | <0.001 |  | 0.002 |
| <2000 | 93(19.7) | 0.00 (0.00) |  | 0.55 (0.50) |  | 0.65 (0.48) |  |
| 2000-5000 | 161(34.1) | 0.03 (0.17) |  | 0.70 (0.46) |  | 0.79 (0.41) |  |
| 5000-10000 | 134(28.4) | 0.04 (0.19) |  | 0.87 (0.34) |  | 0.84 (0.37) |  |
| >20000 | 84(17.8) | 0.12 (0.33) |  | 0.86 (0.35) |  | 0.85 (0.36) |  |
| **First time of colorectal polyps** |  |  | 0.969 |  | 0.248 |  | <0.001 |
| Yes | 376(79.7) | 0.04 (0.20) |  | 0.73 (0.44) |  | 0.82 (0.38) |  |
| No | 96(20.3) | 0.04 (0.20) |  | 0.79 (0.41) |  | 0.64 (0.48) |  |
| **Hyperlipidemia** |  |  | 0.263 |  | 0.122 |  | <0.001 |
| No | 409(86.7) | 0.05 (0.21) |  | 0.76 (0.43) |  | 0.82 (0.39) |  |
| Yes | 63(13.3) | 0.02 (0.13) |  | 0.67 (0.48) |  | 0.56 (0.50) |  |
| **Diabetes** |  |  | 0.465 |  | 0.006 |  | <0.001 |
| No | 344(72.9) | 0.05 (0.21) |  | 0.78 (0.42) |  | 0.83 (0.38) |  |
| Yes | 128(27.1) | 0.03 (0.17) |  | 0.66 (0.48) |  | 0.66 (0.47) |  |
| **Constipation** |  |  | 0.915 |  | 0.166 |  | 0.012 |
| No | 428(90.7) | 0.04 (0.20) |  | 0.75 (0.43) |  | 0.80 (0.40) |  |
| Yes | 44(9.3) | 0.05 (0.21) |  | 0.66 (0.48) |  | 0.64 (0.49) |  |
| **Fatty liver** |  |  | 0.084 |  | 0.524 |  | <0.001 |
| No | 413(87.5) | 0.04 (0.19) |  | 0.75 (0.43) |  | 0.81 (0.39) |  |
| Yes | 59(12.5) | 0.08 (0.28) |  | 0.71 (0.46) |  | 0.58 (0.50) |  |
| **Family members with colorectal polyps** |  |  | 0.075 |  | 0.446 |  | 0.001 |
| No | 301(63.8) | 0.03 (0.17) |  | 0.73 (0.44) |  | 0.83 (0.38) |  |
| Yes | 171(36.2) | 0.06 (0.25) |  | 0.77 (0.42) |  | 0.70 (0.46) |  |
| **Education about colorectal from a hospital** |  |  | <0.001 |  | 0.082 |  | <0.001 |
| No | 291(61.7) | 0.00 (0.06) |  | 0.72 (0.45) |  | 0.84 (0.37) |  |
| Yes | 181(38.3) | 0.10 (0.31) |  | 0.79 (0.41) |  | 0.70 (0.46) |  |

**Table S2. Model fit index**

| **Indicators** | **Reference** | **Results** |
| --- | --- | --- |
| RMSEA | <0.08 | 0.160 |
| SRMR | <0.08 | 0.095 |
| TLI | >0.80 | 0.805 |
| CFI | >0.80 | 0.864 |

**Table S3. Analysis of direct and indirect effects**

| **Model paths** | | Total effects | | Direct effect | | Indirect effect | |
| --- | --- | --- | --- | --- | --- | --- | --- |
|  |  | β(95%CI) | P | β(95%CI) | P | β(95%CI) | P |
| Attitude |  |  |  |  |  |  |  |
|  | Knowledge | 0.586 (0.510,0.662) | <0.001 | 0.586 (0.510,0.662) | <0.001 |  |  |
| Practice |  |  |  |  |  |  |  |
|  | Knowledge | -0.036 (-0.129,0.057) | 0.450 | -0.334 (-0.452, -0.215) | <0.001 | 0.298 (0.208,0.388) | <0.001 |
|  | Attitude | 0.508 (0.388,0.628) | <0.001 | 0.508 (0.388,0.628) | <0.001 |  |  |
